# Supplementary material for: Identification of a Novel Pathogen of Peanut Root Rot, Ceratobasidium sp. AG-A, and the Potential of Selected Bacterial Biocontrol Agents
Source: J Fungi (Basel). 2025 Jun 21;11(7):472. doi: 10.3390/jof11070472 (PMC12295459; doi:10.3390/jof11070472)
Supplement: Supplementary file 1 [file jof-11-00472-s001.zip › jof-3627908-supplementary.pdf]

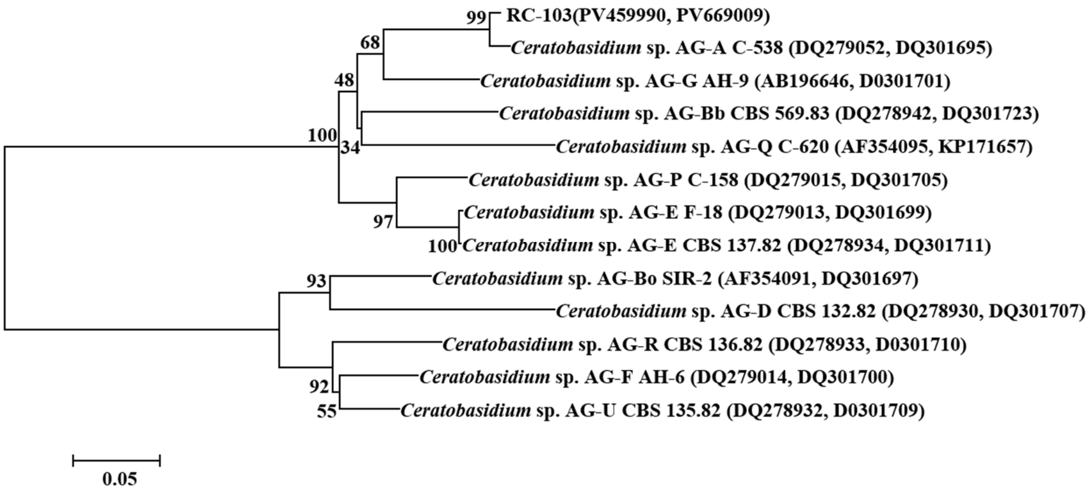

Figure S1 Phylogenetic tree of isolate RC-103 based on sequences of ITS and *RPB2* gene regions. The GenBank accession number for each strain has been provided in the parentheses.

Table S1 Information on three biocontrol bacteria

Strain	Species	Sequences(GenBank access)	Deposit number	Source	District
LY-1	*Bacillus subtilis*	OP199048, OP221239	CGMCC No.23930	The rhizosphere soilwild mint	Qingdao
ZHX-7	*Bacillus velezensis*	MZ474622, MZ484688	CGMCC No.20374	The rhizosphere soil of peanuts	Qingdao
Bc-HN1	*Burkholderia cepacian*	PV683256	CGMCC No.28060	peanut root	Hainan
